# Percutaneous Aortic Balloon Valvuloplasty and Intracardiac Adrenaline in Electromechanical Dissociation as Bridge to Transcatheter Aortic Valve Implantation

**DOI:** 10.1097/MD.0000000000001061

**Published:** 2015-07-02

**Authors:** Jawad Chaara, Pascal Meier, Christophe Ellenberger, Yvan Gasche, Karim Bendjelid, Stephane Noble, Marco Roffi

**Affiliations:** From the Division of Cardiology (JC, PM, SN, MR), Department of Medical Specialties; Division of Anesthesiology (CE); and Division of Intensive Care (YG, KB), Department of Anesthesiology, Pharmacology and Intensive Care, Geneva University Hospitals, Geneva, Switzerland.

## Abstract

This report describes an emergent balloon aortic valvuloplasty (BAV) procedure performed under cardiopulmonary resuscitation in a 79-year-old man with severe symptomatic aortic stenosis (mean gradient 78 mm Hg, valve area 0.71 cm^2^, and left ventricular ejection fraction 40%) awaiting surgery and who was admitted for heart failure rapidly evolving to cardiogenic shock and multiorgan failure. Decision was made to perform emergent BAV. After crossing the valve with a 6 French catheter, the patient developed an electromechanical dissociation confirmed at transesophageal echocardiography and cardiac arrest. Manual chest compressions were initiated along with the application of high doses of intravenous adrenaline, and BAV was performed under ongoing resuscitation. Despite BAV, transoesophageal echocardiography demonstrated no cardiac activity. At this point, it was decided to advance a pigtail catheter over the wire already in place in the left ventricle and to inject intracardiac adrenaline (1 mg, followed by 5 mg). Left ventricular contraction progressively resumed and, in the absence of aortic regurgitation, an intraaortic balloon pump was inserted. The patient could be weaned from intraaortic balloon pump and vasopressors on day 1, extubated on day 6, and recovered from multiorgan failure. In the absence of neurologic deficits, he underwent uneventful transcatheter aortic valve implantation on day 12 and was discharged to a cardiac rehabilitation program on day 30. At 3-month follow-up, he reported dyspnea NYHA class II as the only symptom.

This case shows that severe aortic stenosis leading to electromechanical dissociation may be treated by emergent BAV and intracardiac administration of high-dose adrenaline. Intracardiac adrenaline may be considered in case of refractory electromechanical dissociation occurring in the cardiac catheterization laboratory.

## INTRODUCTION

Percutaneous balloon aortic valvuloplasty (BAV) was first performed in 1986^1^ as an alternative for surgical aortic valve replacement (SAVR) in high-risk patients. First observational studies demonstrated hemodynamic improvement following BAV, but high rate of early restenosis and a considerable rate of complications.^2–5^ Nowadays, several studies^6,7^ have described the feasibility of BAV as a bridge to SAVR or transcatheter aortic valve implantation (TAVI) in patients with cardiogenic shock^8^ or major comorbities,^9^ and current guidelines of the European Society of Cardiology recommend this procedure as a class IIb, level of evidence C for this indication.

The first TAVI procedure was performed in 2002 by Cribier et al^10^ and since then it has become a widely used technique for patients with severe aortic stenosis not suitable for surgery or at high surgical risk. Accordingly, the PARTNER Trials^11,12^ demonstrated the superiority of TAVI over BAV, and standard therapies in inoperable patients and the US CoreValve pivotal trial^13^ showed for the first time superiority of TAVI to SAVR in patients at increased surgical risk.

## CASE REPORT

A 79-year-old patient known for severe symptomatic aortic stenosis (transaortic peak velocity 5.7 m/s, mean gradient 78 mm Hg, and valve area 0.71 cm^2^) awaiting TAVI presented with acute onset of shortness of breath because of heart failure. Prior investigation reported a moderately reduced left ventricular ejection fraction (40%) without significant coronary artery disease documented on coronary angiography. His comorbidities consisted of hypertension, diabetes, chronic alcohol consumption, atrial fibrillation, and chronic renal failure. The logistic EurosSCORE was calculated at 25% for perioperative mortality, while Society of Thoracic Surgeons (STS) risk score was 5%.

**FIGURE 1:**
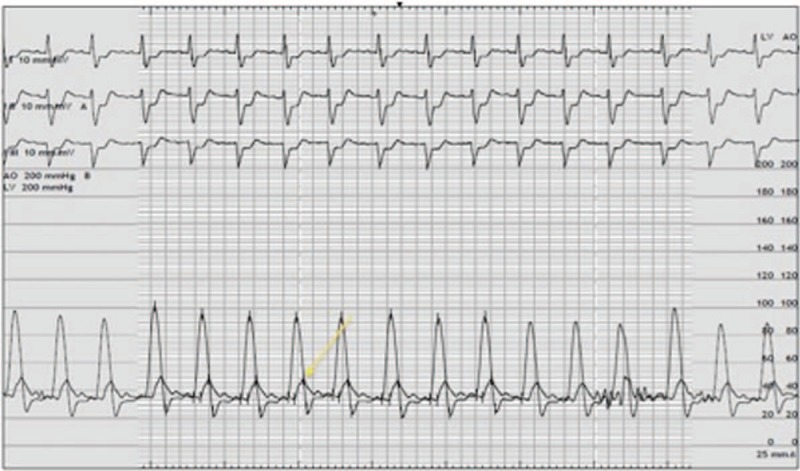
Invasive hemodynamic waves of simultaneous left ventricle–aortic pressures before balloon aortic valvuloplasty showing severe aortic stenosis and cardiogenic shock. Patient conditions degradated following the catheter insertion in the left ventricle. Note the systolic blood pressure of approximately 50 mm Hg (yellow arrow), the left ventricular systolic pressure of approximately 90 mm Hg, and the left ventricular end-diastolic pressure of approximately 35 mm Hg.

**FIGURE 2:**
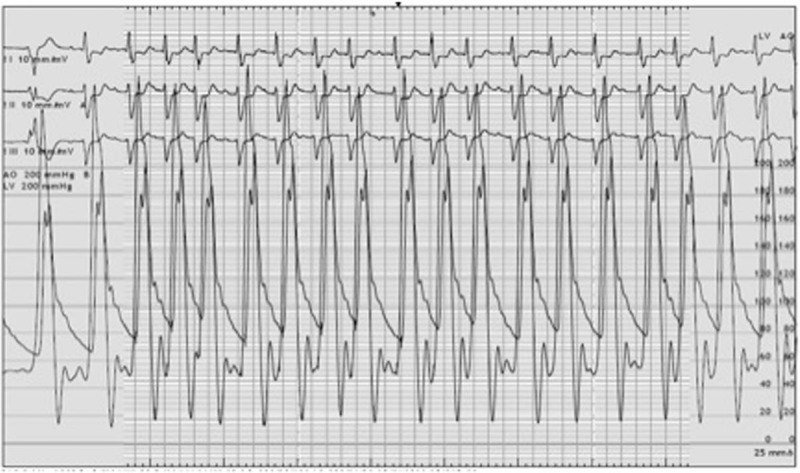
Recovery of hemodynamic parameters after balloon aortic valvuloplasty and intracardiac adrenaline.

Upon presentation, the condition of the patient rapidly deteriorated requiring endotracheal intubation and high doses of cathecholamines, and he developed multiorgan failure. Decision was made to perform percutaneous BAV as a bridge to aortic valve replacement. Arterial vascular access was obtained through transradial and transfemoral approaches for simultaneous pressure measurements and cardiac imaging consisted of both fluoroscopy/angiography and transoesophageal echocardiography (TEE). Following valve crossing, a 6 French pigtail catheter was advanced in the left ventricle, while the second pigtail catheter was positioned in the aortic root and the hemodynamics of the patient further deteriorated. Simultaneous pressure measurement documented systolic aortic pressure of 40 mm Hg and systolic pressure in the left ventricle of 100 mm Hg despite high dose of cathecholamines (Figure 1). TEE excluded cardiac tamponade, but revealed a progressive decrease in contractility and finally electromechanical dissociation, as confirmed by the transesophageal echocardiography probe already in place. Under manual cardiopulmonary resuscitation (CPR), BAV was performed with a 20 mm × 4 cm Nucleus balloon (NuMED, NY), but this led to no contraction of the left ventricle despite high doses of adrenaline administered IV. Over the wire present in the left ventricle, a pigtail was advanced and intracardiac adrenaline (1 mg followed by additional 5 mg) was administered. Cardiac function and blood pressure gradually recovered (Figure 2). In the absence of significant aortic regurgitation, an intraaortic balloon pump was inserted, leading to a further improvement of the hemodynamics. The next day, the inotropes could be weaned off and the intraaortic balloon pump was removed. The patient was extubated on day 6 and organ functions recovered.

Twelve days later, in the absence of neurologic deficits and after a heart team agreement, decision of percutaneous approach was made because of the comorbidities and high intraoperative surgical risk of the patient. The patient underwent a successful transfemoral TAVI via a left femoral approach with a 31 mm CoreValve (Medtronic Inc, MN). Owing to a complete atrioventricular block, this patient with preexisting right bundle branch block required a definitive pacemaker implantation. The patient could be discharged to rehabilitation on day 30 in dyspnea NYHA class II. At 3-month follow-up, he reported dyspnea NYHA class II as the only symptom.

## DISCUSSION

This case report describes the performance of BAV under CPR condition in a patient with severe aortic stenosis as a bridge to TAVI. Despite the lack of left ventricular contraction documented after BAV on TEE, the administration of intracardiac adrenaline followed by intraaortic balloon pump insertion allowed for restoration of cardiac function, stabilization of hemodynamics, and ultimately a staged TAVI procedure.

### BAV as a Bridge to TAVI/SAVR

In patients not immediately suitable for TAVI/SAVR because of hemodynamic instability or multiorgan failure, BAV as a bridge to TAVI/SAVR is a well-described procedure, which demonstrated its feasibility and safety with good midterm outcomes. Indeed, in a large trial^6^ of BAV including 538 BAV procedures, BAV as a bridge was performed in 7% of patients who were in cardiogenic shock, all with good hemodynamic outcomes and without major complications. In addition, literature reported a study that randomized 86 patients at high risk with severe aortic stenosis to undergo BAV as a bridge to TAVI versus no previous BAV, suggesting that performing BAV to relieve symptoms before^14^ TAVI is a safe procedure.

### BAV/TAVI Under CPR

Although no reports on BAV under CPR conditions were identified, 1 previous report describes an emergent TAVI under CPR. Accordingly, Jensen et al^15^ described a CoreValve implantation under Automatic-CPR using the Lucas® Chest Compression System for a severe aortic regurgitation developed during balloon aortic dilatation before TAVI procedure with no major complications at 30-day follow-up.

### Intracardiac Adrenaline for Cardiac Arrest

Adrenaline is a sympathomimetic drug, which helps to improve cardiac output through its action in cardiac B-adrenergic receptors, and its use in intravenous administration is well known in cardiac arrest. However, intracardiac injection was only described in immediate cardiac resuscitation in the 90 seconds by direct injection into right ventricle through a transthoracic parasternal approach. No recent literature has been found on intracardiac injection of adrenaline via a percutaneous catheter in patients with cardiac arrest.

## CONCLUSION

This case shows that electromechanical dissociation in a patient with severe aortic stenosis and cardiogenic shock was reversed by BAV and intracardiac adrenaline and allowed for a bridge to TAVI. Intracardiac adrenaline may be considered in case of refractory electromechanical dissociation occurring in the cardiac catheterization laboratory.

## References

[1] Cribier A Savin T Saoudi N Percutaneous transluminal valvuloplasty of acquired aortic stenosis in elderly patients: an alternative to valve replacement? *Lancet* 1986; 1:63–67. 2867315 10.1016/s0140-6736(86)90716-6

[2] Bashore TM Berman AD Davidson CJ Percutaneous balloon aortic valvuloplasty. Acute and 30-day follow-up results in 674 patients from the NHLBI Balloon Valvuloplasty Registry’. *Circulation* 1991; 84:2383–2397. 1959194 10.1161/01.cir.84.6.2383

[3] Otto CM Mickel MC Kennedy JW Three-year outcome after balloon aortic valvuloplasty. Insights into prognosis of valvular aortic stenosis. *Circulation* 1994; 89:642–650. 8313553 10.1161/01.cir.89.2.642

[4] Davidson CJ Harrison JK Leithe ME Failure of balloon aortic valvuloplasty to result in sustained clinical improvement in patients with depressed left ventricular function. *Am J Cardiol* 1990; 65:72–77. 2294684 10.1016/0002-9149(90)90028-y

[5] Shareghi S Rasouli L Shavelle DM Current results of balloon aortic valvuloplasty in high-risk patients. *J Invasive Cardiol* 2007; 19:1–5. 17297175

[6] Ben-Dor I Maluenda G Dvir D Balloon aortic valvuloplasty for severe aortic stenosis as a bridge to transcatheter/surgical aortic valve replacement. *Catheter Cardiovasc Interv* 2013; 82:632–637. 23015369 10.1002/ccd.24682

[7] Eltchaninoff H Durand E Borz B Balloon aortic valvuloplasty in the era of transcatheter aortic valve replacement: acute and long-term outcomes. *Am Heart J* 2014; 167:235–240. 24439985 10.1016/j.ahj.2013.10.019

[8] Moreno PR Jang IK Newell JB The role of percutaneous aortic balloon valvuloplasty in patients with cardiogenic shock and critical aortic stenosis. *J Am Coll Cardiol* 1994; 23:1071–1075. 8144770 10.1016/0735-1097(94)90592-4

[9] Kogoj P Devjak R Bunc M Balloon aortic valvuloplasty (BAV) as a bridge to aortic valve replacement in cancer patients who require urgent non-cardiac surgery. *Radiol Oncol* 2014; 48:62–66. 24587781 10.2478/raon-2013-0078 PMC3908849

[10] Cribier A Eltchaninoff H Bash A Percutaneous transcatheter implantation of an aortic valve prosthesis for calcific aortic stenosis: first human case description. *Circulation* 2002; 106:3006–3008. 12473543 10.1161/01.cir.0000047200.36165.b8

[11] Leon MB Smith CR Mack M Transcatheter aortic-valve implantation for aortic stenosis in patients who cannot undergo surgery. *N Engl J Med* 2010; 363:1597–1607. 20961243 10.1056/NEJMoa1008232

[12] Smith CR Leon MB Mack M Transcatheter versus surgical aortic-valve replacement in high-risk patients. *N Engl J Med* 2011; 364:2187–2198. 21639811 10.1056/NEJMoa1103510

[13] Adams DH Popma JJ Reardon MJ Transcatheter aortic-valve implantation with a self-expanding prosthesis. *N Engl J Med* 2014; 370:1790–1798. 24678937 10.1056/NEJMoa1400590

[14] Ussia GP Capodanno D Barbanti M Balloon aortic valvuloplasty for severe aortic stenosis as a bridge to high-risk transcatheter aortic valve implantation. *J Invasive Cardiol* 2010; 22:161–166. 20351386

[15] Jensen PB Andersen C Nissen H Transcatheter aortic valve implantation in a patient with circulatory collapse, using the LUCAS(R) chest compression system. *Catheter Cardiovasc Interv* 2013; 81:1084–1086. 23436538 10.1002/ccd.24590

